# The Myth of Formality in the Global North: Informality‐as‐Innovation in Dutch Governance

**DOI:** 10.1111/1468-2427.12706

**Published:** 2019-01-29

**Authors:** Rivke Jaffe, Martijn Koster

**Keywords:** Informality, governance, policy, innovation, Amsterdam, The Netherlands

## Abstract

Why has urban informality in the global North received so little attention? We suggest that this neglect can be explained in part by the tendency of scholarship to reproduce the myth of Northern formality: the widely held belief that informality occurs only in corrupt and clientelist ‘developing countries’. This myth has allowed activities and connections that would generally be framed as clientelist or corrupt in the global South to be rebranded as policy innovation in Western Europe and North America. In this brief paper, we challenge the myth of Northern formality by focusing on two empirical cases of informality in Dutch governance that demonstrate how the state frames the toleration and deliberate use of informality as policy innovations. Specifically, we focus on strategic, uncodified and non‐transparent deviation from legal procedure in order to achieve compliance and/or effectiveness. Relying on ethnographic methods and secondary sources, we discuss firstly the governance of Amsterdam's red light district and secondly participatory infrastructure projects in the surrounding province of North Holland. The first case highlights the strategic non‐enforcement or non‐application of laws, while the second case points to the use of personalized relationships and non‐transparency in participatory governance.

## Introduction

As the editors of this forum argue in their introduction, informality is ubiquitous in cities across the world. Until recently, however, the study of urban informality has tended to focus on cities in the global South. This neglect, we suggest, might be explained by the fact that many urban scholars working in Western Europe and North America have tended to reproduce the stories that their governments like to tell: that these countries and their cities are governed in a formal fashion—if informality was ever a prevalent mechanism of governance here, it is a thing of the past, which now only occurs in corrupt and clientelist ‘developing countries’. While these widely held beliefs contrast with the reliance of the same governments on various informal methods of governance, this myth has allowed activities and connections that would generally be framed as clientelist or corrupt in the global South to be rebranded as indications of policy innovation in the global North.

In this brief paper, we challenge this myth of Northern formality by focusing on two empirical cases of informality in Dutch governance that demonstrate how the state frames the toleration and use of informality as policy innovations. While in the global South such informal governance practices would be taken as evidence of corruption, collusion or a failure of the rule of law, in the Netherlands we see such practices framed as positive innovations that other countries would do well to emulate. Specifically, we focus on the strategic, uncodified and non‐transparent deviation from legal procedure in order to achieve compliance and/or effectiveness.

Relying on ethnographic methods and secondary sources, we discuss firstly the governance of Amsterdam's red light district (RLD) and secondly participatory infrastructure projects in the surrounding province of North Holland. For the RLD, we focus on the longstanding Dutch ‘toleration policy’ (*gedoogbeleid*) that allows for state management and regulation of illegal activities such as drug use and prostitution, and on more recent policy efforts to ‘clean up’ the district, which again rely on informal and semi‐legal mechanisms. In the province of North Holland we concentrate on informal practices in participatory projects related to infrastructure, and specifically on so‐called kitchen table negotiations between residents and state representatives. The first case highlights the strategic non‐enforcement or non‐application of laws that continue to be applied elsewhere, including a reliance on legal instruments that give the authorities increased ‘room for manoeuvre’, while the second case points to the use of personalized relationships and non‐transparency in procedures formally aimed at public, transparent forms of participatory governance.

## Informal governance as ‘innovative’

Informality is often defined as that which is not regulated by the state. In addition, it is generally associated with low‐income marginalized populations. However, as authors such as Ananya Roy (2009) and Colin McFarlane (2012) show based on their work in urban India, elites and government actors also draw strategically on unregulated modes of urban planning and development. Showing how informality is embedded in formal governance arrangements, Daniel Goldstein (2016: 7), in his research on urban Bolivia, uses the term ‘disregulation’ to describe situations where ‘the state administers its own preferred forms of regulation while ignoring others, privileging a system of discretionary surveillance and enforcement’.

Informal practices and arrangements are part and parcel of the workings of the state (Schoon and Altrock, 2014). This insight complicates the dichotomy between the formal and the informal, in which the formal is often used as a synonym for everything designed and controlled by the state, while the informal implies all that is ‘non‐state’ (everything that takes place ‘in the shadows of’ the state or outside state control). Contrary to prevalent analyses, informal practices and arrangements do not only take place outside of officially sanctioned procedures. Rather, they may form an inseparable part of these procedures.

As the introduction to this forum sets out, analysing urban informality requires engaging with how states are imagined. As authors within political sociology and the anthropology of the state have argued, understanding how states work involves studying both everyday bureaucratic practices and the representations that allow ‘the state’ to appear as separate from society (Abrams, 1988; Gupta, 1995). As the editors of this forum point out, the dominant focus on informality in cities of the global South connects to both scholarly and on‐the‐ground imaginaries of the postcolonial state, which is generally understood as ‘less‐than’ (European and American ideal types). These imaginaries of the state are often dystopian, with a lack of state regulation framed as dangerously chaotic, for instance in depictions of ungovernable ‘fragile’ cities (Muggah, 2014). Only rarely is the informality associated with a ‘weak’ or ‘fragile’ state presented in a more positive light, for instance in Rem Koolhaas’ (2002) idea of chaotic urban self‐organization as the dynamic future of cities.

The lack of focus on informality in countries such as the Netherlands is driven by an unrealistic academic and vernacular imagination of the state. Many urban and regional studies of Europe and North America are based (either implicitly or explicitly) on the idea that governance is achieved primarily through formal practices, ties and networks. If informality—such as personalized and non‐transparent transactions, together with unregulated economic activities—is not framed as a thing of the past, it is understood as a marginal presence associated with pockets of poverty or immigrant groups. The conflation of informality and marginality is strengthened by the skew in the literature on informality in the global North towards low‐income and immigrant populations, as indicated by the editors of this forum in their introduction. This assumption of formality has obscured the prevalence of discretionary state ‘disregulation’ and of the personalization of governance practices in Europe and North America (in contrast to the deregulation and privatization described in many studies of the neoliberal or enabling state).

In this paper, we seek to relocate concepts and approaches that were developed primarily in South Asia and Latin America to Western Europe. As our Dutch cases show, a broad range of governance domains have been disregulated, with state actors strategically tolerating and utilizing informality by enforcing legislation and regulation only partially. This mode of governance also emphasizes bureaucratic flexibility and discretion in everyday encounters and problem solving, and allows for explicitly personalist practices, ties and networks. However, this informality is rarely described as such and is hardly ever connected to similar processes in the global South. Rather, in post‐welfare states such as the Netherlands, these informal governance practices have been pitched as policy ‘innovation’. Resembling Swyngedouw's (2005) ‘governance‐beyond‐the‐state’, these policies allow non‐state actors (criminal organizations, corporations, citizens) to negotiate a larger role in domains where state actors are unwilling or unable to enforce stringent regulation. However, as our case of the RLD shows, successive Dutch governments were quick to frame informality as innovation well before their general turn to neoliberal policies in the 1990s.

## Partial unrule of law

The red light district is one of Amsterdam's most famous tourist attractions and is often seen as exemplary of Dutch policies on prostitution and drug use.^1^ These have long been understood locally and internationally through the idea of regulated ‘toleration’ policy, or *gedoogbeleid* in Dutch.^2^ From the 1960s until around the turn of the century, much of such policy could be characterized as something in between legalization and criminalization. While prostitution was not fully legalized until the late 1990s, it was national policy not to prosecute. The idea behind *gedoogbeleid* was largely pragmatic: it is more efficient and effective to contain and regulate certain formally illegal activities without applying criminal law. The head of the Amsterdam police in the mid‐1970s was quoted as explaining that ‘If we were to act exactly according to the law, we would have to abolish the entire sex work industry and everything that comes with it … but we also have to adjust to developments in society’.^3^

In addition to generating various ‘grey’ mechanisms for managing illegal goods and services, this toleration policy has also relied on informal connections between police, policymakers and (suspected) criminals. In the 1970s and 1980s, sex industry entrepreneurs and pimps kept the district relatively safe in order to prevent police intervention and to ensure that clients would find their way to sex workers. In an oral history interview, a former police officer explained that sex industry leaders preferred not to call the police when their customers made trouble; instead, they would employ Hell's Angels members to act as informal policing agents. With the proliferation of hard drugs (especially heroin) during these decades, the population of sex workers came to include more vulnerable groups of drug addicts and immigrants.

A parliamentary inquiry held in the mid‐1990s showed that a select number of organized crime groups were involved not only in prostitution and drug trafficking, but also in human trafficking and money laundering. They ran the local economy, regulating labour in the prostitution sector as well as in cafés, real estate and security, operating what amounted to their own informal zoning laws and licensing system (Brants, 1998: 627–8). In interviews, both police and entrepreneurs confirmed the existence of an informal banking system, in which locals could access unregulated loans, albeit at a monthly interest rate of 25%. Arguably, the ‘pragmatic’ policy of selective non‐enforcement amounted to a semi‐strategic production of variegated sovereignty (*cf*. Ong, 2006), a *de facto* outsourcing of control of the district to the vice industry. This divestment of responsibility took place in a highly opaque fashion.

Starting in the 1990s, consensus began to form that *gedoogbeleid* had resulted in a number of excesses in the RLD, and that the area needed to be ‘cleaned up’. This culminated in the municipal government's ‘Project 1012’, named after the RLD postal code, introduced in 2007 as a comprehensive plan aimed at crime reduction and economic upgrading. This plan involves a reformulation by the municipal government of the governance of the RLD, which relies on ‘innovative’ informal and semi‐legal measures to concentrate sex work and drug‐related activities in a shrinking area and promote state‐led gentrification.

Within the framework of ‘Project 1012’, the municipality began to buy up and renovate properties that were used for prostitution and other ‘low‐value’ or ‘criminogenic’ functions, and to introduce property zoning to promote ‘valuable’ activities such as boutiques and chic coffee bars. In certain cases, property owners are expropriated; in other cases, financial subsidies are used to stimulate entrepreneurs to adapt their business according to this new zoning. In addition, ‘integrity screenings’ have been introduced that bar both convicted and merely suspected criminals from owning or operating ‘undesirable’ businesses. This strategy involves a highly flexible use of legality in its application of administrative law rather than criminal law. Unable to successfully prosecute major sex industry players, state actors draw on spatially delineated legislation to harass both suspected and convicted criminals, and to deny them permits to run legal but allegedly criminogenic businesses. At the same time, the property buy‐outs have resulted in millions of euros of government money being transferred to alleged criminals.

While not uncontroversial, such measures are rarely framed as ‘corrupt’ or ‘undermining the rule of law’—framings that, in the global South, would be applied to comparable cases of public‐to‐private money flows or the state harassment of antagonists. Rather, the municipal government defends these measures as necessarily creative solutions to an intractable governance problem. Where in discussions of ‘developing countries’, Dutch politicians or bureaucrats might refer to informal strategies in order to delegitimize foreign governance actors, here they make reference to such measures to legitimize their own authority. Like the earlier *gedoogbeleid* policies in the RLD, ‘Project 1012’ is presented as a pragmatic and innovative approach, seeking to reconcile a commitment to social order and the rule of law with the reality of crimes and criminals that are hard to prosecute. Both approaches to the RLD entail semi‐official zoning arrangements that involve the production of variegated sovereignty in order to facilitate contradictory state objectives—a modality of informal rule comparable to that described by Picker (2019, this forum) in his analysis of NGO rule in Romani camps in Montreuil.

## Informal participatory governance

A very different case of informality in state practices is found in a number of participatory projects conducted by the province of North Holland, in which the city of Amsterdam is located.^4^ In contrast to municipal governments in the Netherlands, citizen participation is new to provincial government, the administrative level between national and municipal government. The research on which this case is based studied two participatory infrastructural projects: one aimed at the improvement of a provincial road, the other at dike reinforcement. Both projects followed a specific set of regulations aimed at guaranteeing citizen participation.

In the road improvement project, provincial bureaucrats organized multiple participatory meetings. In these meetings, the provincial government invited residents who lived along the road or in the villages that were connected by the road to discuss and vote on multiple scenarios. Since all of the scenarios necessitated additional land, the province had to expropriate property from several farmers and other landowners. In what they presented as tailor‐made negotiations, the bureaucrats invited all owners to what the local area manager referred to as ‘kitchen table conversations’. One of the bureaucrats, responsible for contact with citizens, said: ‘It takes place simply at the farm, at the kitchen table … Sometimes [the land] has a whole history. It belonged to their grandfather or it has been family property for hundreds of years. Sometimes there are all kinds of family feuds’. He explained that he had to solve these problems at the kitchen table and find a reasonable solution.

The bureaucrat in charge of the taskforce that monitors and promotes citizen participation in the provincial government explained that it was critical to hold kitchen table conversations prior to the start of the official participatory trajectory: ‘Before we start with the real citizen participation, we know who the stakeholders are … We need to go and talk to these people’. Other bureaucrats explained that if landowners were to refuse the final offer for their land, they could be expropriated through a lengthy legal process, since road improvement, like dike reinforcement, could be framed as a ‘collective security issue’ in which the authorities had a final say. Such kitchen table conversations also took place in the dike reinforcement project, especially with the owners of dike houses. Here the issue was not so much expropriation, but the possible impact in terms of real estate value that might lead to costly lawsuits.

The idea of tailor‐made negotiations, which included discussing the non‐monetary meanings of land, granted bureaucrats greater discretionary power. It represented a strategy to move from a legal procedure of expropriation towards a personalized relationship. The bureaucrats involved saw the negotiations as a way to achieve effective outcomes, framing the quality of state–citizen relations in terms of legitimacy rather than legality. This personalization and the emphasis on intimate spaces of interaction were especially important, as distrust of government officials was known to generate resistance and result in lengthy and expensive court cases contesting expropriation. Landowners themselves acknowledged their appreciation of this approach.

The kitchen table negotiations were a widely employed practice, accepted by all parties involved. These household‐level negotiations were included as a standard phase in the official trajectories of the participatory approach, taking place before and during collective participatory meetings with all residents. The general, formal procedure of citizen participation was open to the public, transparent and documented in detail. In contrast, these kitchen table conversations were held privately in domestic settings and were not documented anywhere. In so doing, they formed an opaque element in participatory procedures intended to open up governance to citizens. Although similar informal practices have often been labelled as ‘clientelist’ and ‘corrupt’ in studies on the lack of ‘good governance’ in the global South (see e.g. Khan and Swapan, 2013), in this context they were considered to be a contribution to novel forms of citizen participation.

## Conclusion

This brief paper has focused on the uses of informality in Dutch governance, drawing on two cases that illustrate how informality is embedded in the official procedures and projects of the state. Both cases demonstrate how the authorities strategically depart from legally transparent and codified procedures, adopting opaque and personalist mechanisms of governance in order to enhance the efficiency and effectiveness of particular policies. In so doing, both cases show how formal governance arrangements are entangled with informal practices. Personal contacts between citizens, (illegal) entrepreneurs and government representatives play a decisive role in these governance processes. This analysis connects to work by Ananya Roy and others, who emphasize the strategic use of informal practices by states, but shows how in the Netherlands such practices are not seen as shady power moves or ‘less‐than’ modes of governance. Rather, state agents claim and promote these practices—in all apparent sincerity—as laudable policy innovations, sometimes referring explicitly to a supposedly Dutch tradition of creative pragmatism. This successful, legitimizing definition of ‘informality‐as‐innovation’ by institutional actors is key to understanding why popular and academic perceptions of Dutch governance rarely involve ‘informality‐as‐a‐problem’.

The Netherlands is often seen—not least by its own citizens—as a highly organized and regulated society. We suggest that this pervasive, normative and often self‐congratulatory ‘myth of formality’ has deflected the attention of informality researchers away from recognizing the pivotal role that informal practices and arrangements play in such ‘formal’ contexts. Debunking this myth, and correcting this epistemological skew in studies of urban and regional governance, involves attending more closely to informal practices, even in seemingly highly formalized places. The rethinking of mainstream analyses of European and North American governance is facilitated by mobilizing and translating concepts developed in, for instance, South Asia and Latin America—rethinking the conceptual geographies of informality becomes easier when we challenge dominant geographies of theory.

## Footnotes

1 This paper draws on research conducted by Rivke Jaffe with the assistance of Marthe Singelenberg (University of Amsterdam), based on secondary sources, media analysis and oral history interviews with former and current residents, police officers, entrepreneurs and workers in the sex industry, and drug addicts.

2 This resembles a particular type of Schoon and Altrock's (2014: 216) ‘conceded informality’ in present‐day China, where the state tolerates selected informal practices.

3 H. Korver, ‘Ouders laten 8‐jarig dochtertje naakt fotograferen: Activiteiten sex‐koning Jan B. worden uitvoerig nagetrokken’ [‘Parents allow 8‐year‐old daughter to be photographed naked: thorough investigation of sex‐king Jan B.’s activities’], *De Telegraaf*, 12 April 1975 (author translation).

4 Between September 2014 and July 2015, Martijn Koster coordinated this research on participatory governance, for the Province of North Holland, carried out with Restlan Aykaç (Province of North Holland) and Olga Verschuren (Utrecht University).
